# Effect of COVID-19 response work experience on turnover intention among employees of dedicated COVID-19 hospitals in Seoul

**DOI:** 10.1186/s12960-024-00926-9

**Published:** 2024-06-13

**Authors:** Eunyoung Park, Chang Hoon You, Hyojee Joung, Young Dae Kwon

**Affiliations:** 1 Department of Healthcare Policy, Seoul Public Health Policy Institute, Seoul Medical Center, Seoul, Korea; 2 Department of Public Health, Graduate School of Public Health, Seoul National University, Seoul, Korea; 3 Department of Humanities and Social Medicine, College of Medicine, The Catholic University of Korea, 222 Banpo-Daero, Seocho-gu, Seoul, 06591 Korea; 4 Catholic Institute for Public Health and Healthcare Management, The Catholic University of Korea, Seoul, Korea

**Keywords:** Intention to leave, COVID-19 dedicated hospital, Hospital staff

## Abstract

**Background:**

According to previous studies, stress and job burnout among medical personnel increased during the COVID-19 pandemic. This study analyzed the effect of the experience of COVID-19 response work on the intention of municipal hospital staffs to leave their workplaces during the pandemic.

**Methods:**

The 3556 employees who had worked for more than 1 year at one of the eight Seoul Municipal Hospitals that either provided inpatient treatment for quarantined COVID-19 patients or operated as screening clinics were taken as the study population. In total, 1227 employees completed a web or mobile survey between October 21 and November 18, 2020. A chi-squared test was performed to confirm the difference in the distribution of turnover intention depending on whether the employees performed COVID-19 response tasks. Multiple logistic regression analyses were performed to determine the factors that affected the intention to leave.

**Results:**

Of the 1227 respondents, 761 (62.0%) were frontline workers who were the first line of response to COVID-19. Experience with COVID-19 response tasks (OR = 1.59, *p* = 0.003) was significantly associated with the intention to leave. Additionally, the probability of turnover intention was significantly higher among workers aged 20–29 years (OR = 2.11, *p* = 0.038) and 40–49 years (OR = 1.57, *p* = 0.048), unmarried individuals (OR = 1.66, *p* = 0.005), doctors (OR = 2.41, *p* = 0.010), nurses (OR = 1.59, *p* = 0.036), and technical staff members (OR = 2.22, *p* = 0.009). High turnover intention was found among those who experienced high levels of burnout (OR = 2.03, *p* < 0.001) and those working in non-directly managed municipal hospitals (OR = 1.87, *p* = 0.018).

**Conclusion:**

Employees directly involved in COVID-19 response work displayed higher turnover intention. Various personal, job, and organizational factors significantly influenced employees’ intentions to leave their positions in dedicated COVID-19 hospitals. These findings suggest the necessity of introducing management programs to aid workers who have experienced sudden changes in their duties and loss of autonomy while performing COVID-19 response tasks.

## Background

The catastrophic infectious disease outbreak of COVID-19 caused massive physical, social, and economic changes in everyday life. Facilities in our daily lives, such as schools, restaurants, cafés, parks, gyms, and libraries, underwent a variety of changes. Among these, hospitals, the frontlines of defense against COVID-19, were required to make significant changes in their spatial, systematic, and operational methods. In particular, in hospitals that were designated as dedicated COVID-19 facilities, in addition to adjusting to substantial facility changes such as the installation of negative pressure rooms, employees also had to contend with adjustments such as wearing protective gear, processes for providing service in isolation wards, and changes in ward layouts and shiftwork systems [1, 2].

In the early days of the COVID-19 outbreak in Korea, the incident rates increased sharply from the epicenters in large cities such as Seoul. Seoul, the capital city, has a tightly packed mass of residents and resources, with a large inflow population from abroad. Around 18.5% of the total population and 12.5% of the country’s sickbeds are concentrated in the capital city. Seoul accounted for 31.3% of the confirmed COVID-19 cases in Korea by the end of 2020. To tackle this severe crisis, Seoul secured municipal hospitals in the city center and dedicated them to the COVID-19 emergency response. The proportion of municipal hospital beds (3816 beds) among all hospital beds (74 931 beds) in Seoul is 5.1%. This is lower than that in other countries such as Japan (22.8%) and the UK (100.0%) but is similar to that of public hospital beds nationwide in South Korea (5.2%). Consequently, municipal hospitals, accounting for only 5.1% of all hospital beds in Seoul, were coping with 70.4% of the total COVID-19 caseloads by the end of 2020. Of the 12 municipal hospitals, eight including “general hospitals” and “hospitals” were converted to operate as COVID-19-dedicated hospitals and screening clinics, excluding four “hospitals” responsible for special areas such as psychiatry and dentistry. A “general hospital” is a hospital with more than 300 beds and nine or more medical departments and treats a wide range of diseases, while a “hospital” is a small or medium sized hospital that provides outpatient and inpatient treatment based on a few medical departments. A municipal hospital refers to a hospital that receives subsidies from Seoul Metropolitan Government. Among these, four directly managed municipal hospitals (DMMHs) are operated directly by the Seoul Metropolitan Government. The employees are public officials, and all financial resources for operation and management are provided by the Seoul Metropolitan Government. The remaining four non-DMMHs are owned by the Seoul Metropolitan Government but are operated as a contracting-out or public enterprise and receive partial subsidies. During this transition period, patients who had previously been admitted to municipal hospitals were transferred to other hospitals. Municipal hospital staff, amidst fears stemming from the infection, also had to deal with the burden of new duties in addition to their existing ones [1, 3].

Extensive environmental and contextual changes affect organizational turnover rates. Stress, depression, and various other problems were reported by a significant number of medical staff during their response to MERS, an infectious disease epidemic that occurred prior to COVID-19 [4]. Of the nurses working during the MERS epidemic, 57.1% reported having post-traumatic stress disorder, stemming from their experience [5]. Recent research conducted during the COVID-19 pandemic also reported increased job exhaustion and turnover intention among hospital employees at the forefront of the COVID-19 response [6, 7]. Many individuals suffered from anxiety, depression, and distress from the experience [3].

Turnover intention is a critical issue in human resource management, particularly in hospitals. Medical staff are highly trained professionals who are difficult to replace quickly because of the significant costs and time required for recruitment, selection, and training [8–11]. The departure of skilled medical staff has a negative impact on organizational efficiency and the quality of medical care [12]. Medical institutions in many countries face staff shortages, which poses a major obstacle to reinforcing healthcare services [10]. Accordingly, several studies have examined turnover intention among medical personnel. The results show that turnover intention is influenced by numerous factors including demographics, job characteristics, and job satisfaction [10, 13, 14]. A systematic review of nurses’ turnover intention found that, in addition to individual and job factors, inter-relational and organizational factors also influence their turnover intention [14, 15]. Furthermore, psychological factors such as job satisfaction and burnout are related to turnover intention [10, 13, 16].

This study aimed to analyze turnover intention based on experience in COVID-19-related duties, and to identify the related influential factors; it focused on the employees of eight municipal hospitals in Seoul that were converted to operate as dedicated COVID-19 hospitals since the outbreak of the pandemic.

## Methods

### Study design and population

To analyze the intention to leave based on experience in COVID-19 response work, a survey was conducted on 3566 employees who had worked for more than a year at eight municipal hospitals, which formed the core medical institutions of Seoul’s COVID-19 response system, from October 21 to November 18, 2020. These eight hospitals comprised four “general hospitals” and four “hospitals” that were either converted to accept only confirmed COVID-19 patients during the pandemic or operated screening clinics while maintaining some of their existing functions. Accordingly, employees who performed COVID-19 response tasks and general medical treatment coexisted within the same hospital. Considering the COVID-19 situation, the survey was remotely implemented through the internet and mobile devices. The sample size of this study was set at 822, based on a 95% confidence interval and 3% standard error. There were 1227 respondents out of 3556 employees to whom the survey was distributed, with a response rate of 34.4%. The difference (15.2–79.2%) in responses was due to variations in hospital size and circumstances. To correct for differences in response rates by hospital, the regression analysis included institutional characteristics, such as hospital size, type, and governance. To increase the response rate, information was sent via text message at least three times, and the survey manager in each hospital encouraged participation.

This study was approved by the Institutional Review Board (IRB) of Seoul Medical Center (approval No. SEOUL 2020-4-026-006). Informed consent was obtained from all participants involved in the study.

### Variables and measurements

To investigate turnover intention, the dependent variable in this study, items from the Turnover Intention Scale-6 (TIS-6), developed by Bothma and Roodt, were used [17]. This scale includes statements such as “I have the intention to leave” and “I am thinking about moving to a hospital that better suits my personal needs.” It comprises six questions addressing the intention to leave and has been widely used as a turnover intention survey for doctors and nurses [18, 19]. Each item was rated on a 5-point Likert scale, and the average of the six questions was used in the analysis. If the average score exceeded 3 points, it was defined as the intention to leave. Cronbach’s alpha, which indicates the internal consistency between items, was 0.84.

A preliminary interview was conducted with the employees of the target medical institutions to design the questionnaire and identify the variables influencing turnover intention. Based on the results of previous research and preliminary interviews, personal factors (sex, age, marital status, monthly salary, and education level), job factors (COVID-19 response work, period of employment, nature of occupation, job burnout, and job satisfaction), and organizational factors (hospital size, governance, and type) were examined to determine their impact on turnover intention. Regarding personal factors, age groups were classified as 20–29 years, 30–39 years, 40–49 years, and > 50 years old. Marital status was divided into married and unmarried, and unmarried included those who are single (never married), divorced, or widowed. Monthly salary was divided into less than three million Korean won (2499 dollars), three to five million (2499 to 4165 dollars), and over five million (4165 dollars). Considering that most medical personnel are highly educated, education level was categorized into those who had completed graduate school and those who had not.

Regarding job factors, an affirmative response to the question “Did you participate in direct response to COVID-19 at the hospital?” was used to determine whether participants had performed COVID-19 response duties. Direct response tasks included working in negative-pressure isolation rooms and isolation wards, working in in-hospital screening clinics, and dispatching out-of-hospital COVID-19 response tasks (e.g., lifestyle treatment centers and epidemiological survey sites). Both the period of employment at the hospital and general career length were investigated and divided into categories of 4 years or less, 5–9 years, 10–14 years, and 15 years or more. Occupations were grouped as doctors, nurses, health service providers, administrators, and technicians. The category of health service providers included pharmacists, nutritionists, radiologists, clinical pathologists, physical therapists, occupational therapists, dental hygienists, and medical recorders. The technicians included mechanical engineers, building engineers, electricians, environmental engineers, computational workers, drivers, security personnel, ward assistants, cooks, and cleaners.

The Maslach Burnout Inventory (MBI) was used to assess job burnout. This inventory includes a separate survey for medical staff; however, in this study, the general occupational group survey tool (MBI-GS) was used as it targeted various occupations within the hospitals. The MBI consists of 16 questions; however, based on its Korean translation and the results of a validity survey, one question related to cynicism was removed, resulting in a 15-item version [20]. Items were rated on a 7-point Likert scale (0–6 points), and the average score for each item was used in the analysis. Job burnout was defined as having an emotional exhaustion score of 3.2 points or more, a cynicism score of 2.4 or more, or a professional efficiency score of 3.8 or less, based on an analysis of the median job burnout criteria in previous studies. To assess job satisfaction, items from the K-Public Hospital Job Satisfaction Tool, developed for public hospital employees, were used [21]. There are 29 items in total, consisting of questions related to duties (4 items), internal communication (4 items), evaluation and compensation (4 items), work environment (3 items), safety and employee grievances (6 items), satisfaction with the hospital (3 items), and overall satisfaction (5 items). Each item was measured on a 5-point Likert scale, and the average of the seven questions domain was used in the analysis. A job satisfaction score of 3 or higher was defined as high satisfaction.

Organizational factors included hospital size, governance, and type. Hospitals were divided into two groups: those with more than 500 beds and those with fewer than 500 beds. Hospital governance was divided into DMMHs, where all employees were public officials and whose budgets and operations were managed within the public organization system, and non-DMMHs, which were either operated by special corporations or were private contract hospitals funded by the city administration. Hospital types were classified as “hospitals” and “general hospitals.”

### Statistical analysis

Chi-squared tests were performed to confirm the distribution according to the participants’ characteristics, and to confirm the difference in the turnover intention distribution depending on whether the participants performed COVID-19 response tasks. Multiple logistic regression analyses were performed to determine the factors affecting turnover intention. At this point, the COVID-19 response work status as well as personal, job, and organizational factors were included. All analyses were performed using SAS version 9.4 (SAS Institute Inc., Cary, NC, USA), and the significance level was set at 5% on both sides.

## Results

During the survey period, there were 1227 respondents, of whom 62.0% performed frontline COVID-19 response work. Among them, 75.6% were women, 64.5% were unmarried, and 22.7% had completed graduate school. Doctors, nurses, and health service providers accounted for 7.1%, 52.5%, and 15.7% of the sample, respectively. In addition, 36.1% of respondents worked at hospitals with 500 or more beds, 32.3% were at directly managed municipal hospitals, and 58.0% were at “general hospitals” (Table 1).

**Table 1:** General characteristics of study population

		Total	COVID-19 response work experience		*p-*value of *χ*^*2*^-test
			Frontline	Second-line	
Overall (n, (%))		1227 (100.0)	761 (62.0)	466 (38.0)	
Individual factor (n, (%))					
Sex	Men	299 (24.4)	172 (22.6)	127 (27.3)	
	Women	928 (75.6)	589 (77.4)	339 (72.7)	
Age (year)	20–29	159 (13.0)	123 (16.2)	36 (7.7)	***
	30–39	429 (35.0)	258 (33.9)	171 (36.7)	
	40–49	362 (29.5)	221 (29.0)	141 (30.3)	
	≥ 50	277 (22.5)	159 (20.9)	118 (25.3)	
Marital status	Unmarried	791 (64.5)	472 (62.0)	319 (68.5)	*
	Married	436 (35.5)	289 (38.0)	147 (31.5)	
Education level	≤ Undergraduate	949 (77.3)	573 (75.3)	376 (80.7)	
	Postgraduate	278 (22.7)	188 (24.7)	90 (19.3)	
Monthly salary (1000 KRW)	**≤ **2999	333 (27.2)	207 (27.2)	126 (27.0)	
	3000–5000	393 (32.0)	236 (31.0)	157 (33.7)	
	≥ 5000	501 (40.8)	318 (41.8)	183 (39.3)	
Job-related factor (n, (%))					
Occupation	Doctor	87 (7.1)	72 (9.5)	15 (3.2)	***
	Nurse	644 (52.5)	433 (56.9)	211 (45.3)	
	Technician	135 (11.0)	64 (8.4)	71 (15.2)	
	Administrator	168 (13.7)	72 (9.5)	96 (20.6)	
	Health service provider	193 (15.7)	120 (15.8)	73 (15.7)	
Years in present organization	**≤ **4	539 (43.9)	338 (44.4)	201 (43.1)	
	5–9	329 (26.8)	206 (27.1)	123 (26.4)	
	10–14	156 (12.7)	97 (12.7)	59 (12.7)	
	≥ 15	203 (16.6)	120 (15.8)	83 (17.8)	
Years in present work	≤ 4	245 (20.0)	157 (20.6)	88 (18.9)	
	5–9	301 (24.5)	190 (25.0)	111 (23.8)	
	10–14	241 (19.6)	149 (19.6)	92 (19.7)	
	≥ 15	440 (35.9)	265 (34.8)	175 (37.6)	
Burnout	High	397 (32.4)	258 (33.9)	139 (29.8)	
	Low	830 (67.6)	503 (66.1)	327 (70.2)	
Job satisfaction	High	965 (78.6)	592 (77.8)	373 (80.0)	
	Low	262 (21.4)	169 (22.2)	93 (20.0)	
Organizational factor (n, (%))					
Size of hospital (bed)	≥ 500	443 (36.1)	240 (31.5)	203 (43.6)	***
	**≤ **499	784 (63.9)	521 (68.5)	263 (56.4)	
Governance of hospital	Non-DMMH	831 (67.7)	504 (66.2)	327 (70.2)	
	DMMH	396 (32.3)	257 (33.8)	139 (29.8)	
Type of hospital	“General hospital”	711 (58.0)	406 (53.4)	305 (65.5)	***
	“Hospital”	516 (42.0)	355 (46.6)	161 (34.5)	

^*^p < 0.05, **p < 0.01, ***p < 0.001; KRW: Korean won; DMMH: directly managed municipal hospital

The average score of the question, “How often have you considered leaving your job” was 2.6 ± 1.3, and “How often are you frustrated when not given the opportunity at work to achieve your personal work-related goals?” was 3.0 ± 1.2. Employees who were assigned to the COVID-19 response work (frontline workers) had significantly higher turnover intention than second-line workers (39.7% vs. 28.1%; *p* < 0.001). In the individual factor category, in terms of sex, the turnover intention of frontline workers was higher than that of second-line workers for both male and female participants, but only the female (41.3% vs. 28.9%; *p* < 0.001) participants showed a significant difference. By age, the turnover intention of frontline workers was higher than that of second-line worker in all groups. However, the distribution of turnover intention between the frontline groups and second-line groups was significantly different only in the 40–49 age group (35.3% vs. 23.4%; *p* = 0.017). By marriage status, frontline workers’ turnover intention was high in both the married and unmarried groups, but a significant difference was found only in the unmarried group (52.6% vs. 39.5%; *p* = 0.009). The turnover intention of frontline workers was significantly high in all groups by salary and education levels.

In terms of job-related factors, the turnover intention of frontline workers was high among doctors, nurses, and technicians, but only the nurse group (45.5% vs. 28.4%; *p* < 0.001) was statistically significant. Regarding the year in present organization, the turnover intention of frontline workers was high in all groups but was significantly higher in the ≤ 4 years (41.1% vs. 27.9%; *p* = 0.002) and 5–9 years (46.1% vs. 35.0%; *p* = 0.047) groups. In terms of years in present work, frontline workers’ turnover intention was high in all groups, but was significantly higher only in the ≤ 4 years (46.5% vs. 26.1%; *p* = 0.002) and 10–14 year (41.6% vs. 28.3%; *p* = 0.037) groups. The group with high job burnout showed a large gap in turnover intention compared to that with low job burnout (high group 72.1% vs. low group 25.3%). However, within the group with high job burnout, there was no statistically significant difference in turnover intention depending on frontline work assignment. This can be interpreted as the fact that when job burnout is high, employees already feel a high intention to leave even if they are not assigned to frontline work. Similarly, the group with high job satisfaction had lower intention to leave than that with low job satisfaction (high group 21.5% vs. low group 64.2%). Nevertheless, the group with low job satisfaction showed high turnover intention regardless of whether they were assigned to the frontline; thus, there was no significant difference in distribution depending on whether they were assigned to the frontline. Meanwhile, even in the group with low job burnout (29.6% vs. 18.5%; *p* < 0.001), turnover intention was significantly higher when assigned to frontline work than second-line work. Even in the group with high job satisfaction (25.5% vs. 15.3%; *p* = 0.001), turnover intention was significantly higher when assigned to frontline work.

In terms of organizational factors, the turnover intention of frontline workers was high in all groups by hospital bed but was significantly higher only in the group with ≥ 500 beds (49.2% vs. 24.1%; *p* < 0.001). By governance of hospital, the turnover intention of frontline workers was significantly higher only in non-DMMH hospitals (47.6% vs. 29.7%; *p* < 0.001). By type of hospital, the turnover intention of frontline workers was high in all groups but was significantly higher only in the “general hospital” group (49.5% vs. 29.2%; *p* < 0.001) (Table 2).

**Table 2:** Percentage of turnover intention according to COVID-19 response work experience

		Total (n = 1227)	COVID-19 response work experience		*p-*value of *χ*^*2*^-test
			Frontline (n = 761)	Second-line (n = 466)	
Overall (%)		35.3	39.7	28.1	***
Individual factor (%)					
Sex	Men	30.8	34.3	26.0	
	Women	36.8	41.3	28.9	***
Age (year)	20–29	50.3	54.5	36.1	
	30–39	45.0	47.7	40.9	
	40–49	30.7	35.3	23.4	*
	≥ 50	17.7	21.4	12.7	
Marital status	Married	28.2	31.8	22.9	**
	Unmarried	48.2	52.6	39.5	**
Monthly salary (1000 KRW)	≤ 2999	44.1	48.8	36.5	*
	3000–5000	33.8	38.6	26.8	*
	≥ 5000	30.5	34.6	23.5	**
Education level	≤ Undergraduate	36.6	40.8	30.1	***
	Postgraduate	30.9	36.2	20.0	**
Job-related factor (%)					
Occupation	Doctor	36.8	40.3	20.0	
	Nurse	39.9	45.5	28.4	***
	Technician	29.6	34.4	25.4	
	Administrator	29.8	29.2	30.2	
	Health service provider	28.0	27.5	28.8	
Years in present organization	≤ 4	36.2	41.1	27.9	**
	5–9	42.0	46.1	35.0	*
	10–14	37.2	40.2	32.2	
	≥ 15	20.7	24.2	15.7	
Years in present work	≤ 4	39.2	46.5	26.1	**
	5–9	48.5	52.1	42.3	
	10–14	36.5	41.6	28.3	*
	≥ 15	23.4	25.7	20.0	
Burnout	High	72.1	75.2	66.7	
	Low	25.3	29.6	18.5	***
Job satisfaction	High	21.5	25.5	15.3	***
	Low	64.2	67.4	58.3	
Organizational factor (%)					
Size of hospital (bed)	≥ 500	37.7	49.2	24.1	***
	≤ 499	33.9	35.3	31.2	
Governance of hospital	Non-DMMH	40.6	47.6	29.7	***
	DMMH	24.2	24.1	24.5	
Type of hospital	“General hospital”	40.8	49.5	29.2	***
	“Hospital”	27.7	28.5	26.1	

^*^p < 0.05, **p < 0.01, ***p < 0.001; KRW: Korean won; DMMH: directly managed municipal hospital

Logistic regression analyses revealed that the probability of turnover intention was higher among frontline workers than second-line workers (OR = 1.59, *p* = 0.003). After adjusting for potential covariates, the prevalence of intention to leave was significantly higher among respondents who were 20–29 years old (OR = 2.11, *p* = 0.038) and 40–49 years old (OR = 1.57, *p* = 0.048), as compared to the reference category of ≥ 50. Compared to the reference married group, the turnover intention of the unmarried group was significantly higher (OR = 1.66, *p* = 0.005). In terms of occupation, doctors (OR = 2.41, *p* = 0.010), nurses (OR = 1.59, *p* = 0.036), and technicians (OR = 2.22, *p* = 0.009) were the significant predictors for turnover intention compared to reference group of health service providers. In terms of burnout, the probability of turnover intention was 2.03 times higher for every 1-point increase (*p* < 0.001), and in terms of job satisfaction, the probability of turnover intention decreased by 0.67 times for every 1-point increase (*p* < 0.001) In terms of organizational factors, non-DMMH workers (OR = 1.87, *p* = 0.018) were significantly associated with higher odds of having an intent to leave compared to the reference group of DMMH workers (Table 3).

**Table 3:** A multiple logistic regression analysis of factors associated with turnover intention

		Adjusted OR	95% CI	*p-*value
COVID-19 response work experience	Frontline worker	1.59		**
	Second-line worker (ref)	1.00	1.18–2.15	
Individual factor				
Sex	Women	1.04	0.70–1.53	
	Men (ref)	1.00		
Age (year)	20–29	2.11	1.04–4.26	*
	30–39	2.26	1.34–3.81	**
	40–49	1.57	1.00–2.47	*
	≥ 50 (ref)	1.00		
Marital status	Unmarried	1.66	1.16–2.37	**
	Married (ref)	1.00		
Education level	≤ Undergraduate	1.29	0.87–1.91	
	Postgraduate (ref)	1.00		
Monthly salary (1000 KRW)	**≤ **2999 (ref)	1.00		
	3000–5000	1.01	0.69–1.47	
	≥ 5000	0.82	0.57–1.20	
Job-related factor				
Occupation	Doctor	2.41	1.24–4.70	**
	Nurse	1.59	1.03–2.46	*
	Technician	2.22	1.22–4.03	**
	Administrator	1.18	0.68–2.04	
	Health service provider (ref)	1.00		
Years in present organization	≤ 4	1.16	0.67–2.03	
	5–9	1.14	0.65–2.01	
	10–14	1.50	0.82–2.75	
	≥ 15 (ref)	1.00		
Years in present work	≤ 4	0.73	0.41–1.31	
	5–9	1.15	0.70–1.89	
	10–14	0.87	0.54–1.41	
	≥ 15 (ref)	1.00		
Burnout (MBI-GS)		2.03	1.72–2.39	***
Job satisfaction (K-PHJS)		0.67	0.56–0.82	***
Organizational factor				
Size of hospital (bed)	≥ 500	0.90	0.62–1.31	
	≤ 499 (ref)	1.00		
Governance of hospital	Non-DMMH	1.87	1.11–3.15	*
	DMMH (ref)	1.00		
Type of hospital	“General hospital” (ref)	1.00		
	“Hospital”	1.06	0.62–1.81	

^*^p < 0.05, **p < 0.01, ***p < 0.001; OR: odds ratio; CI: confidence interval; KRW: Korean won; ref: reference; DMMH: directly managed municipal hospital

## Discussion

This study aimed to examine the effect of changes in the hospital work environment on staff members’ turnover intention during the COVID-19 pandemic. This study is significant as a large-scale turnover intention survey on healthcare workers in the early stages of responding to COVID-19. The results confirmed that, while controlling for influencing factors established in previous studies, the experience of COVID-19 response work had a significant effect on turnover intention. According to previous studies, stress and job burnout among medical personnel increased during the COVID-19 pandemic [6]. In particular, workload, dealing with death and dying, personal demands and fears, dealing with strict biosecurity measures, and stigma were major factors that increased the stress levels of medical staff performing COVID-19 response tasks [22]. Changes in duties that altered perceived risk, affected social relationships, and increased workload and job stress were found to affect turnover intention [23]. In addition, factors such as low social support, depression, and having children affected turnover intentions [24, 25]. In this study, owing to various factors, including increased workload, increased stress, increased burden of wearing protective equipment, alienation from family, and increased uncertainty due to the risk of infection, employees who performed direct COVID-19 response tasks showed higher turnover intention than those who performed general duties.

The patterns of job turnover intention differed by occupational group, with doctors showing a particularly high intention to leave. In response to COVID-19, doctors performed various support tasks in addition to working in isolation wards, such as working in screening clinics, lifestyle treatment centers, and dispatching epidemiological investigations. At the hospital level, normal outpatient treatment and surgery could not be performed in hospitals that suspended outpatient treatment and were converted to isolation hospitals. During an interview at a municipal hospital included in this study, a surgeon said, “I am afraid that my hands, which have always operated on patients, will become dull as I will not be able to perform surgeries for a year or more.” In fact, in the early stages of responding to the pandemic, Seoul dedicated more than 90% of the beds in municipal hospitals to COVID-19. During this process, most of the doctors in municipal hospitals, irrespective of their individual will or area of specialization, experienced changes in their work inconsistent with their duties, such as working in isolation wards and screening clinics, and performing epidemiological investigations. Consequently, some municipal hospitals that were converted to COVID-19 dedicated hospitals in 2020 recorded a doctor turnover rate of over 30%, according to the Seoul municipal hospitals annual report. When compared to the results of the 2020 Korean Healthcare Survey, where the average doctor turnover rate was 17.2%, the turnover rate of Seoul municipal hospitals was high.

These findings have important implications for human resource management, to be applied in hospital settings in crisis situations. In crisis situations such as the pandemic, human resource management requires a delicate approach that considers occupation and individual characteristics. Healthcare workers could not choose whether to work in response to COVID-19, and they were randomly assigned to each part according to a policy decision. It is necessary to utilize individual characteristics, such as turnover intention tendencies identified in ordinary times and the characteristics of each occupational group, to determine work division and placement during the emergency period. Such a careful consideration is necessary to avoid collapse of the human resource management system, such as large-scale turnover and manpower shortages in response to emergencies.

Furthermore, the turnover intention for “technical jobs,” including mechanical engineers, building engineers, electricians, environmental engineers, ward assistants, and cleaning staff, was significantly high. As the functions of municipal hospitals changed to those of hospitals dedicated to COVID-19, the affected occupational groups experienced considerable burdens. These ranged from carrying out construction and equipment changes in a short time, such as installing negative pressure wards and air conditioning and circulation reorganization, to the maintenance and operation of converted facilities. Even in the case of back-end support workers not directly dealing with COVID-19 patients, the shift to a dedicated hospital resulted in a significant change in the work environment.

Turnover intention is affected by demographic factors such as age and sex [13, 15, 25]. In this study, the turnover intentions of members aged 20–29 years, who were new to the workforce, and those aged 30–39 years and 40–49 years, who had mid-range experience in the workforce, were higher than those of employees aged 50 years or older. This finding is consistent with the results of previous studies that found high turnover intentions among new employees who were inexperienced in their work [13], and that job pressure and burnout were high for those with experience (preceptors) who were skilled in their duties but performed face-to-face work with patients at the forefront of the COVID-19 response [26]. In this study, the proportion of workers involved in responding to COVID-19 was much higher in the younger age groups. Therefore, the effects of these differences on turnover intention should be considered. Newly hired employees are expected to lack on-the-ground experience. Among the employees aged 20–29, 54.5% of those who were assigned to COVID-19 response work, and 36.1% of those who performed second-line tasks, showed turnover intention. However, the proportion of employees aged 40–49 who performed COVID-19 response work was higher than that of employees aged 50 years or older, and turnover intention among those with experience in response to COVID-19 was higher in those aged 40–49. This is because the relevant age group was in charge of practical fieldwork and carried the burden of directing young employees. In establishing a response system for an emergency, careful consideration is required for middle managers and new employees.

In terms of marital status, the high turnover intention among unmarried workers is consistent with the results of previous studies [10]. Research has shown that unmarried people have weaker social and emotional support infrastructure than those who are married, which is known to have a negative impact on job burnout. Therefore, further analysis of this mechanism in a pandemic situation is needed.

Psychological factors, such as job burnout, stress, and job satisfaction, are expressed in interactions with the workplace environment and influence the turnover intention of medical staff [13, 15, 27, 28]. This study found that higher job burnout was associated with higher turnover intention, whereas higher job satisfaction was associated with lower turnover intention. Therefore, it is important to identify employees with suspected burnout on normal working days and use this as evidence for work assignments during a pandemic or emergency. In response to sudden changes in the work environment, such as conversion to a COVID-19 dedicated hospital, focused methods are known to increase employee satisfaction, such as offering educational programs, implementing appropriate compensation systems, and identifying and improving factors related to job satisfaction. In relation to job burnout, responses such as identifying known stress factors and searching for solutions, as well as identifying those at elevated risk for job burnout and applying a prevention program targeting them, should be implemented soon after any changes in the work environment.

Various changes in “job demands” occurred in COVID-19 dedicated hospitals; however, identifying specific and detailed changes to tasks and workload due to COVID-19 response, as well as investigating the effect of these changes (e.g., in the level of support from superiors, support from colleagues, compensation and organizational support systems) on turnover intention, were beyond the scope of this study. Previous studies have reported that work assignments during the COVID-19 period lead to an increase in workload, resulting in burnout and a decrease in work engagement [29]; therefore, during this period, human resource support is needed for the staff [30]. Follow-up research is needed to analyze the mechanisms of multidimensional changes in duties due to COVID-19 and their effects on turnover intention.

## Limitations

This survey was conducted using a large sample during the COVID-19 response period. However, as this was a cross-sectional study, a baseline for turnover intention was not presented. Consequently, it was not possible to confirm the changes before and after the response to COVID-19. In addition, although the required sample size was met, there is a possibility that selective bias, such as differences in survey response rates by institutions, may have occurred because of the survey and response processes in the extreme situation of responding to COVID-19. In addition, non-responding employees should be considered. As COVID-19 response tasks are assigned more to young employees than older ones, the impact of this factor also needs to be considered.

## Conclusions

In the face of the COVID-19 pandemic, various personal, job, and organizational factors significantly influenced employees’ intentions to leave their positions at COVID-19 dedicated hospitals. Importantly, this study confirmed that employees directly involved in the COVID-19 response work displayed higher turnover intention. These findings indicate the necessity of introducing administrative programs to aid workers who have experienced sudden changes in their duties and loss of autonomy while performing COVID-19 response tasks. Concurrently, to respond to any future calamitous changes in healthcare work environments, it is important to implement improvement activities at the individual and organizational levels, focusing on worker-related and organizational causes of turnover intention.

## Data Availability

Due to individual privacy and ethical constraints, the dataset underlying this analysis cannot be made publicly available. The dataset cannot be completely de-identified without removing key variables such as place of hospital, work years in present organization, position level, occupation, and health status. The datasets used and/or analyzed are available from the corresponding author on reasonable request.

## Abbreviations

COVID-19: Coronavirus disease-19
KRW: Korean won
SD: Standard deviation
DMMH: Directly managed municipal hospital
OR: Odds ratio
CI: Confidence interval
ref: Reference
